# Effects of Chitosan on *Candida albicans*: Conditions for Its Antifungal Activity

**DOI:** 10.1155/2013/527549

**Published:** 2013-06-17

**Authors:** Antonio Peña, Norma Silvia Sánchez, Martha Calahorra

**Affiliations:** Departamento de Genética Molecular, Instituto de Fisiología Celular, Universidad Nacional Autónoma de México, Circuito Exterior s/n, Ciudad Universitaria, 04510 México, DF, Mexico

## Abstract

The effects of low molecular weight (96.5 KDa) chitosan on the pathogenic yeast *Candida albicans* were studied. Low concentrations of chitosan, around 2.5 to 10 **μ**g*·*mL^−1^ produced (a) an efflux of K^+^ and stimulation of extracellular acidification, (b) an inhibition of Rb^+^ uptake, (c) an increased transmembrane potential difference of the cells, and (d) an increased uptake of Ca^2+^. It is proposed that these effects are due to a decrease of the negative surface charge of the cells resulting from a strong binding of the polymer to the cells. At higher concentrations, besides the efflux of K^+^, it produced (a) a large efflux of phosphates and material absorbing at 260 nm, (b) a decreased uptake of Ca^2+^, (c) an inhibition of fermentation and respiration, and (d) the inhibition of growth. The effects depend on the medium used and the amount of cells, but in YPD high concentrations close to 1 mg*·*mL^−1^ are required to produce the disruption of the cell membrane, the efflux of protein, and the growth inhibition. Besides the findings at low chitosan concentrations, this work provides an insight of the conditions required for chitosan to act as a fungistatic or antifungal and proposes a method for the permeabilization of yeast cells.

## 1. Introduction

Chitosan is an N-deacetylated derivative of chitin, mostly extracted from exoskeletons of crustacea; it is biodegradable, biocompatible, and with low toxicity, which makes it useful and convenient in many different practical applications, such as those in water and waste treatment, food and beverages, cosmetics and toiletries, industrial processes, textile, paper and agriculture industry, biopharmaceutics, biotechnology, and medical uses, mostly based on its antimicrobial and antifungal properties among others [1, 2].

Antimicrobial properties of cationic polymers have been known for a long time; the first studies revealed the permeabilization of yeast cells as their main effects [3]. Later on, cytochrome *c* and diethylaminoethyl-dextran were used to permeabilize the yeast cell membrane [4, 5]. Some studies have demonstrated the disruption of bacterial membranes, both by chitosan itself and by some derivatives [6]. These effects have also been documented using three *Candida* species [7]. In bacteria, polycationic chitosan can interact with the negatively charged membrane and alter bacterial surface morphology, which either increases membrane permeability, causing leakage of intracellular substances, or decreases membrane functions, preventing nutrient transport [8]. Chitosan effects have not been fully studied, and its direct utilization as an antimicrobial agent is limited by its low solubility at neutral pH [2].

Several other cationic compounds are known to produce an efflux of K^+^ in yeast [9]. In plants, the efflux of ions (K^+^, H^+^, Ca^2+^, and Cl^−^) has also been observed [10]. Also a K^+^ leakage has been identified in *Glycine max* [11] and in fungi, like *Sphaeropsis sapinea *and* Trichoderma harzianum*. These fluxes may alter the ionic balance needed to maintain the principal functions of the organisms [12].

It has been suggested that at a pH below 6.3, chitosan molecules become positively charged by protonation of the amino groups that interact with the negative charges of the cell surface. It was also found that blocking the amino groups prevents the antimycotic properties of chitosan [13].

Considering that *Candida albicans *is an opportunistic pathogen and infections with this fungus are a serious problem, particularly in immune-compromised patients, experiments reported were performed with this yeast, but also with its closely related species, *Candida dubliniensis* [14], as well as with *Saccharomyces cerevisiae*.

This paper describes the effects of chitosan: a large efflux of K^+^ and other small molecules, a remarkable increase of Ca^2+^ uptake, and the inhibition of respiration, fermentation, and viability. However, in complex medium, high concentrations of chitosan are required to inhibit growth. A proposal of the mechanism by which chitosan may act as an antifungal, as well as its possible use to study enzymes and metabolic functions *in situ*, and compartmentalization of solutes are included.

## 2. Materials and Methods

### 2.1. Strains and Growth Conditions

Most studies were performed with *C. albicans* strain ATCC 10231, reported by the American Type Culture Collection as isolated from a patient with bronchomycosis. The *S. cerevisiae* strain was a commercial diploid strain isolated from a single colony (La Azteca, SA, México). Some experiments were performed with *C. dubliniensis* strain CD 36. Besides, considering that our results with the *C. albicans* strain 10231 might be due to its particular properties, some experiments were repeated with the strain ATCC 90028, where indicated. This strain is reported by the ATCC as isolated from human blood.

Cells were maintained in solid YPD medium (1% yeast extract, 2% bactopeptone, 2% glucose, and 2% agar), renewing the cultures once a month. A loophole of cells from the solid medium was placed into 500 mL of medium without agar. After growth in an orbital shaker at 29°C for 24 hours, the cells were collected by centrifugation, washed twice with water, and suspended in water. Then they were either used during the same day or fasted by suspending them in 250 mL of water and aerating for 48 h in the orbital shaker at 250 rpm, at 29°C. *S. cerevisiae *was starved 24 h, but *C. albicans* required 48 h to reach almost the same starvation state [15]. After collection and washing by centrifugation, the cells were suspended in water to a ratio of 0.5 g·mL^−1^ (wet weight, working suspension) and maintained in ice until their use during the same day. From the initial experiments, it was found that the effects of different concentrations of chitosan varied depending on the amount of cells, in most experiments, 12.5 mg·mL^−1^, wet weight was used.

### 2.2. Potassium Efflux and Acidification

Potassium efflux and acidification of the medium were followed by means of pH and K^+^ selective electrodes connected to a pH meter and an adequate acquisition system. Usually, 125 mg wet weight of starved cells (12.5 mg·mL^−1^) as incubated in 2 mM MES-TEA, pH 6.0, and 20 mM glucose, in a final volume of 10.0 mL. While following the tracings and at the indicated times, variable amounts of chitosan were added as indicated in the figures. Experiments were carried out in a chamber with continuous magnetic stirring, maintained at 30°C. 

### 2.3. Efflux of Molecules at Higher Chitosan Concentrations

Yeast cell membrane permeabilization by chitosan was examined by measuring the efflux of material absorbing at 260 nm, K^+^, and phosphate. Effluxes were measured by incubating 50 mg (12.5 mg·mL^−1^) of starved cells in 10 mM MES-TEA, pH 6.0, and 20 mM glucose, for 10 min at 30°C in a final volume of 4.0 mL. After incubation with variable concentrations of chitosan, the cells were centrifuged, and the supernatant was used to measure K^+^ with a flame photometer, material absorbing at 260 nm (nucleotides) with a spectrophotometer, and inorganic phosphate by the method of Fiske and Subbarow [16]. Total K^+^ of the cells was extracted by boiling a similar suspension of cells in a water bath for 20 min. Total inorganic phosphate or material absorbing at 260 nm was obtained by a 20 min extraction in ice of a similar amount of cells with 30% trichloroacetic acid.

### 2.4. Glucose-6-phosphate Dehydrogenase Activity

To examine the yeast cell membrane permeability to proteins produced by chitosan, as well as the integrity of the internal enzymes, glucose-6-phosphate dehydrogenase activity was measured both in the supernatant of the samples incubated with variable concentrations of chitosan, or with a similar incubation mixture with whole cells to which variable concentrations of chitosan were added. The measurements were done in 10 mM MES-TEA, pH 6.0, 600 *μ*M glucose-6-phosphate, 500 *μ*M NADP^+^, and 12.5 mg·mL^−1^ of cells in a final volume of 2.0 mL. Absorbance changes were measured at 340 nm in an Olis converted DW 2a UV/VIS AMINCO spectrophotometer.

### 2.5. Plasma Membrane Potential Difference Estimation

The plasma membrane potential difference was estimated as described before [17] following the fluorescence changes of DiSC_3_(3). Starved cells (12.5 mg·mL^−1^) were added to the spectrofluorometer cell containing 10 mM MES-TEA buffer, 20 *μ*M CCCP, 10 *μ*M BaCl_2_, and 200 nM cyanine, in a final volume of 2.0 mL. Where indicated, either 10 mM glucose or 10 mM KCl, or variable amounts of chitosan was added. The fluorescence changes at 540–590 nm, for excitation and emission, respectively, were followed against time in an Olis converted SLM AMINCO spectrofluorometer, equipped with a thermostated cell holder at 30°C under continuous stirring.

### 2.6. Zeta Potential

This parameter was obtained from the displacement velocity of starved cells under an electric field and measured directly under the microscope in a Zeta Meter ZM-75 cell electrophotemeter, which allows calculation of the electrophoretic mobility. From this last parameter, the zeta potential can be obtained by the following Helmholtz-Smoluchowski equation:

(1) $$\begin{array}{c} \text{Zeta}\;\;\text{potential}=\text{EM}\;\;4\pi \;\frac{V_{t}}{D_{t}}, \end{array}$$

where

- EM is the electrophoretic mobility;
- *V*_*t*_ is the viscosity of the liquid measured in poises, at the temperature of the measurement;
- *D*_*t*_ is the dielectric constant of the suspending fluid at the temperature of the measurement;
- 4*π* = 12.57.

The medium (13 mL final volume) contained 10 mM MES-TEA buffer, 2 mM glucose, 12.5 mg of cells (wet weight), and different amounts of chitosan. The electric potential difference applied was 100 V.

### 2.7. ^86^Rb^+^ and ^45^Ca^2+^ Uptake

Uptake of  ^86^Rb^+^ or ^45^Ca^2+^ was measured by adding 12.5 mg of starved cells (25 mg·mL^−1^) to 10 mM MES-TEA, pH 6.0, and 20 mM glucose, 0.5 mL final volume, plus the indicated amounts of chitosan in a water bath at 30°C. After 2 min, 0.1 to 0.6 mM of ^86^RbCl or 50 *μ*M ^45^CaCl_2_ was added. After two more minutes, aliquots of 0.1 mL were taken. They were filtered through a 0.25 *μ*m nitrocellulose filter and washed twice with 0.5 mL of 10 mM CaCl_2_. The filters were then dried and placed in vials with scintillation liquid and counted in a Beckman scintillation counter.

### 2.8. Effects on Cell Viability

To explore this parameter, in 96-well plastic plates and using a multichannel pipette, 5 *μ*L of the nonstarved working suspension (2.5 mg of cells) and tenfold successive dilutions was added to 200 *μ*L (12.5 mg·mL^−1^) of 10 mM MES-TEA, pH 6.0 plus the following additions: (1) 20 mM glucose; (2) 20 mM glucose and 10 mM KCl; (3) 110 mM glucose; (4) 20 mM glucose and 2% bactopeptone; (5) 20 mM glucose with 1% yeast extract; or (6) 200 *μ*L of YPD. In each case, the indicated amount of chitosan was added, and the cells were incubated for 10 min at 30°C, under shaking. After incubation, the corresponding yeast suspensions at the same time were spotted in solid YPD medium, using a solid pin multiplate replicator. Growth was observed after 48 hours. 

Also, colony forming units (CFUs) were determined after incubation of *C. albicans*, with chitosan at variable concentrations under different conditions. Nonstarved cells (12.5 mg·mL^−1^) were incubated for 10 min at 30°C in 10 mM MES-TEA buffer, pH 6.0 plus the additions indicated in the previous experiment, or in YPD, in a final volume of 1 mL. Then, 5 *μ*L of a 10,000-fold dilution (approximately 200 cells) was spread in solid YPD medium, and colonies were counted after 48 h.

Growth curves were performed in a Bioscreen C instrument. For that purpose, after growing the cells in YPD for 24 h as usual, they were suspended at a final optical density of 0.03 units at 600 nm in 300 *μ*L of YPD medium, with variable amounts of chitosan (5, 10, 25, 50, 100, 200, 300, and 400 *μ*g, equivalent to 3.33 times those values, as *μ*g·mL^−1^). Microplates were continuously shaken, and the optical density of each well was measured at 600 nm every 20 min for 48 hours at 30°C. 

### 2.9. Chitinase Assay

Cells were grown as for the growth curves mentioned before, with variable amounts of chitosan (25, 50, 100, 200, 400, 600, and 800 *μ*g, equivalent to 3.33 times those values, as *μ*g·mL^−1^). Samples were taken after 10 h, 24 h, and 48 h and centrifuged. Supernatants were used to measure chitinase activity using Chitinase assay kit from Sigma-Aldrich (Cat. number CS0980-1KT) as recommended by the manufacturers.

### 2.10. Oxygen Consumption

It was measured with a Clark electrode connected to a polarization and measurement device (Yellow Springs), collecting the data with a computer. The incubation medium was 10 mM MES-TEA buffer in a final volume of 5.0 mL, with 20 mM glucose as substrate. Tracings were started with 25 mg (wet weight) (5 mg·mL^−1^) of starved cells to follow the oxygen consumption rate. The experiments were conducted in a closed chamber maintained at 30°C under continuous stirring. In one set of experiments, a mixture of 20 mM potassium phosphate, 1.4 mM NAD^+^, 1.8 mM ATP, and 5 mM MgCl_2_ was also added. Variable amounts of chitosan were added as indicated in the figures.

### 2.11. Fermentation

Ethanol production was measured by incubating 12.5 mg of starved cells, wet weight, in a final volume of 1.0 mL containing 10 mM MES-TEA, pH 6.0, 20 mM glucose, and variable amounts of chitosan as indicated in Figure 11. Incubation was performed in test tubes in a water bath at 30°C. After 10 min, the tubes were cooled in an ice water mixture and centrifuged. Ethanol measurements were performed in aliquots of the supernatants with an Ethanol Kit (Boehringer Mannheim/R-Biopharm. Enzymatic Bio Analysis) as recommended by the manufacturers.

### 2.12. Binding of Chitosan to the Cells

Cells (12.5 mg, wet weight) were incubated for 10 min at 30°C in 10 mM MES-TEA buffer, pH 6.0, or 2% bactopeptone; or in 1% yeast extract; or in YPD, plus the indicated additions of chitosan (final volume 1 mL), and then centrifuged in a bench centrifuge (1625 g) to measure the chitosan remaining in the supernatant. The method consisted in measuring the fluorescence increase (350–440 nm, excitation-emission wavelengths, resp.) of a 10 *μ*M solution of calcofluor white 28 (Sigma Cat. number F-3397) in 2.0 mL of a 10 mM bicine-TEA buffer, pH 8.0. To that purpose, a 1 mM stock solution of the dye was prepared in dimethylsulfoxide. It is to be noted that after the addition of the dye to the measuring mixture, a very large fluorescence was observed, which decayed with time; so, it was let under the light until it attained a low and steady value of the fluorescence. Then, in successive, alternate additions, concentrations of  1 *μ*g·mL^−1^ of chitosan or 20 *μ*L of the supernatant obtained after incubation and centrifugation of the cells were added to the spectrofluorometer cell to record the fluorescence increases. From the relative values of the fluorescence increases resulting from standard additions and those of the supernatant, the remaining concentration of chitosan in the supernatant after incubation could be calculated.

Chitosan (low molecular weight) was obtained from Sigma-Aldrich (Cat. number 448869) and dissolved at 10 mg·mL^−1^ of water to which lactic acid was added to reach a pH around 4.5. From this stock solution, other dilutions were prepared for the experiments. Molecular mass was determined by mass spectrometry and found to be 96.5 kDa.

All experiments were performed at least three times, although in some cases, only representative data are presented.

## 3. Results

### 3.1. Permeability Changes

**Figure 1:**
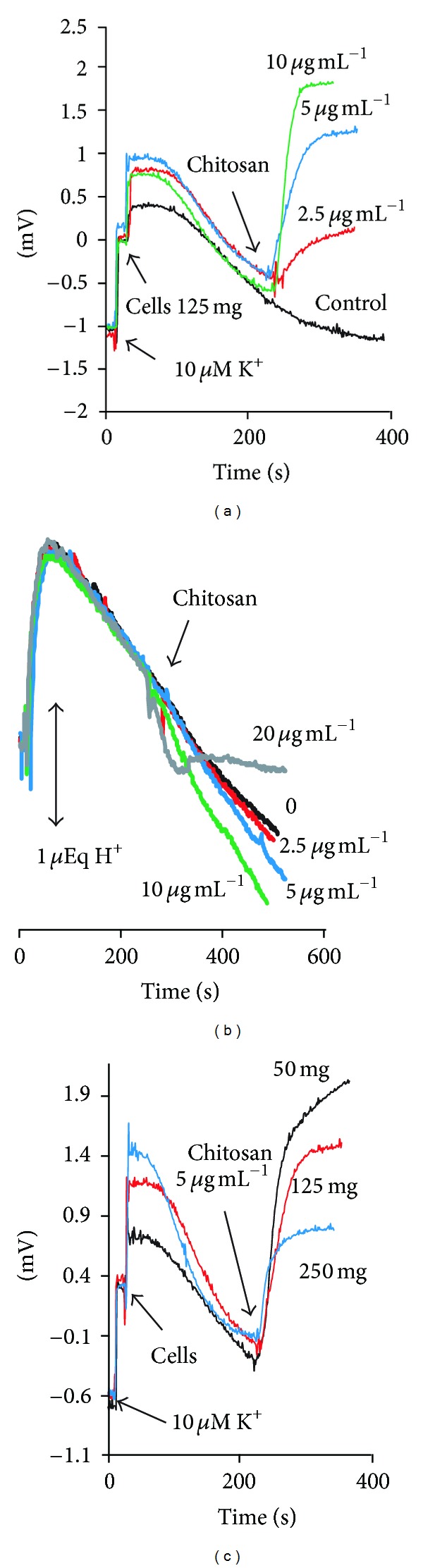
Efflux of K^+^ and effects on acidification of *C. albicans* strain ATCC 10231 produced by variable concentrations of chitosan. In (a) and (b), the incubation medium was 2 mM MES-TEA buffer, pH 6.0, and 20 mM glucose. At the start of the tracings, 10 *μ*M KCl was added as a concentration reference, followed by starved cells (125 mg, wet weight). After the uptake of K^+^ was complete, the indicated concentrations of chitosan were added. In (b), the tracings of the acidification of the medium are shown. In (c), different amounts of cells were added as indicated with a constant chitosan concentration.

Chitosan mostly being a glucosamine polymer, a strongly cationic compound, our first consideration was that it might have effects similar to that of other cationic polymers, like those reported by Yphantis et al. [3], or diethylaminoethyl-dextran, increasing the permeability of the plasma membrane [4, 9, 11]. In preliminary experiments, we found that low concentrations of chitosan, around 2.0 *μ*g·mL^−1^ in 10 mL of incubation medium produced the efflux of significant amounts of K^+^ from a commercial strain of *S. cerevisiae* (not shown). So, we decided to investigate whether in *C. albicans* it also produced the efflux of K^+^ and changes of pH of the medium with selective electrodes. In these experiments, the concentration of 12.5 mg·mL^−1^ of cells was maintained. At the starting of the tracings, as a reference, we added 10 *μ*M KCl; the addition of the cells produced another small increase of the potassium ion concentration of the medium, followed by its uptake to the initial levels or lower. Then, the addition of chitosan (2.5, 5.0, or 10 *μ*g·mL^−1^) produced a clear efflux of the cation (Figure 1(a)), as well as an increase of the acidification rate of the medium (Figure 1(b)). Large chitosan concentrations produced an inhibition and reversal of the acidification of the medium.

It was found that the effect of chitosan depended on the amount of cells used. Using a lower amount of cells, a proportionally larger efflux was observed (Figure 1(c)), indicating a strong binding of the polymer.

**Figure 2:**
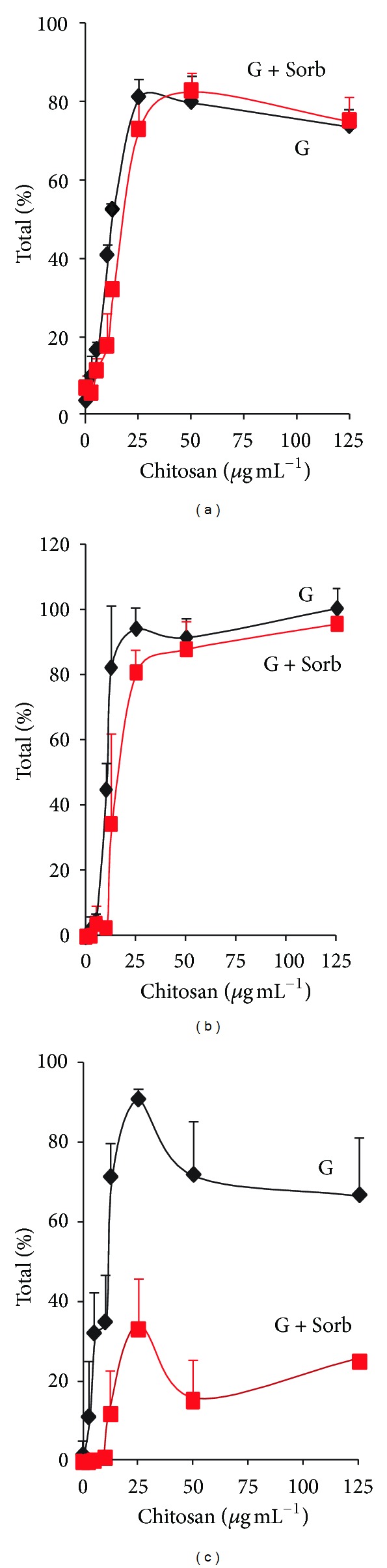
Efflux of nucleotides (material absorbing at 260 nm), K^+^, and phosphate produced by chitosan at variable concentrations on the strain* C. albicans* ATCC 10231. Cells (50 mg, wet weight) were incubated for 10 min at 30°C in 10 mM MES-TEA, pH 6.0, and 20 mM glucose, with (G + Sorb) or without (G) 0.8 M sorbitol, in a final volume of 4.0 mL and the indicated concentrations of chitosan. After incubation, the cells were centrifuged, and the different materials were measured in the supernatant as described under experimental procedures. (a) Material absorbing at 260 nm; (b) potassium, and (c) phosphate.

In view of the limited sensitivity range of the electrode, experiments were performed incubating the cells for 10 min in test tubes with a wider concentration range of chitosan, and after centrifugation, analyzing the supernatant for nucleotides (material absorbing at 260 nm), K^+^, and phosphate. Results are shown in Figure 2; the concentrations of the three parameters in the supernatant showed an increase that depended on the chitosan concentration. 

Since these effects indicated the permeabilization of the plasma membrane, it was also considered interesting adding 0.8 M sorbitol with the idea of exploring the integrity of the internal compartments. The experiments showed that when the cells were incubated with sorbitol the efflux of nucleotides and potassium was similar in its absence or presence (Figures 2(a) and 2(b)), but it significantly prevented phosphate efflux (Figure 2(c)). 

### 3.2. Glucose-6-phosphate Dehydrogenase

After incubating cells with low or moderate concentrations of chitosan, no absorbance peak at 280 nm in the supernatant was detected; so, we decided to measure the activity of glucose-6-phosphate dehydrogenase directly in whole cells in the presence of variable amounts of chitosan. This was performed with the idea that even though proteins at intermediate concentrations of chitosan did not leak out, small molecules such as NADP^+^ and glucose-6-phosphate might enter the cells and reach the enzyme inside, allowing to find out about its integrity. Incubating whole cells with chitosan, activity was detected when the amount of chitosan reached 50 *μ*g·mL^−1^ in the assay mixture. In the supernatants after incubation with chitosan, the activity of the enzyme could only be detected in those from cells previously incubated with 250 *μ*g·mL^−1^ chitosan or higher, indicating that in fact (a) the disruption caused by chitosan on the cell membrane did not result in leakage of enzymes out of the cell but allowed the entrance of small molecules (substrates), and (b) much higher concentrations of chitosan were required to allow the efflux of proteins (not shown). This experiment also showed that even though the plasma membrane had been disrupted at intermediate or moderate chitosan concentrations, enzymes remaining inside could be studied *in situ*.

### 3.3. The Plasma Membrane Potential

**Figure 3:**
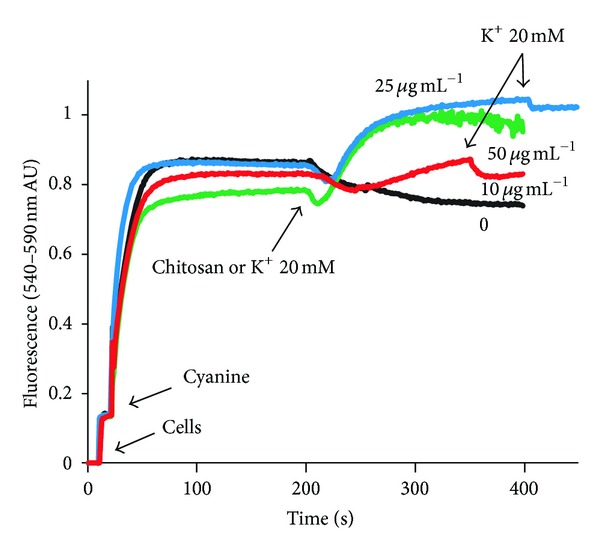
Effects of chitosan on the estimated electric plasma membrane potential difference on the strain* C. albicans* ATCC 10231. The cells (25 mg) were added to the spectrofluorometer cell containing 10 mM MES-TEA buffer, 20 *μ*M CCCP, 10 *μ*M BaCl_2_, and 20 mM glucose, in a final volume of 2.0 mL. After 30 s, 200 nM of the cyanine DiSC_3_(3) was added. Where indicated, 20 mM KCl or variable amounts of chitosan was added. The fluorescence changes, 540–590 nm, for excitation and emission, respectively, were followed. AU: arbitrary units.

K^+^ efflux appeared to be accompanied by the measured anions (nucleotides and inorganic phosphate); however, the possibility that chitosan produced an electrogenic efflux of the cation was explored. For this purpose, the effects of the polymer were studied by estimating the membrane potential by the fluorescence changes of DiSC_3_(3). This method can be used in this yeast, as shown by the fluorescence increase observed when adding glucose to starved cells, as well as by the decrease produced by the addition of K^+^ on this parameter [17]. It was found (Figure 3) that the addition of chitosan in fact produced an increase of the fluorescence, in agreement with what was suspected, that is, that the efflux of K^+^ is electrogenic and hyperpolarizes the plasma membrane. The results of these experiments showed a fluorescence increase at all concentrations used, although at the highest ones, the tracings became noisy because of the flocculation of cells.

### 3.4. Z Potential

**Figure 4:**
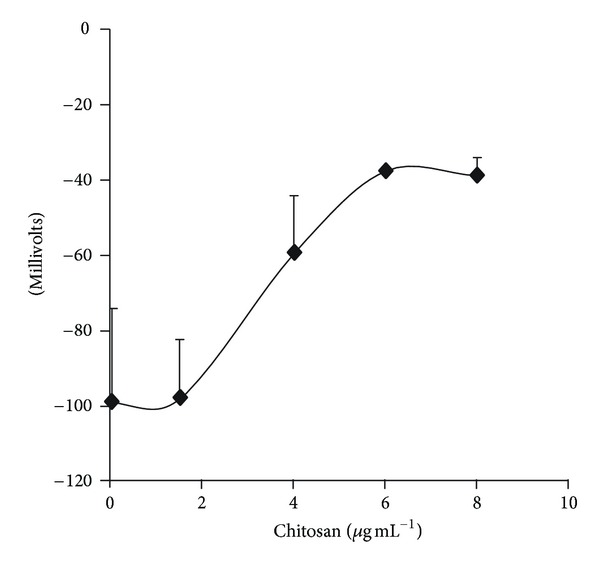
Effects of chitosan on the *ζ* potential of *C. albicans* ATCC 10231 cells. The mobility of the cells was measured against time in a Zeta Meter ZM-75 cell electrophotemeter, to calculate the electrophoretic mobility. From this last parameter, the zeta potential was obtained by the Helmholtz-Smoluchowski equation. The medium (13 mL final volume) contained 10 mM MES-TEA buffer, 2 mM glucose, 12.5 mg of cells (wet weight), and the indicated amounts of chitosan. The potential difference applied was 100 V.

Considering that chitosan might bind to the cell surface due to its positive charge and modify it, we measured the zeta potential as an indicator of the electric surface charge. The effects of chitosan on the zeta potential, as calculated from their mobility in the electrophotemeter, revealed that increasing concentrations of chitosan within the range used in other experiments produced a clear decrease of this parameter (Figure 4). 

### 3.5. Uptake of  ^86^Rb^+^ and ^45^Ca^2+^

The possible mechanisms considered for the efflux of K^+^ produced by chitosan were that (a) the neutralization of the surface charge may decrease the actual concentration of K^+^ in the vicinity of its transporter (s) resulting in its efflux through a carrier similar or different to that used for the uptake, and (b) it may be due to the stimulation of the H^+^/cation antiporter already described by Krauke and Sychrova [18]. The second possibility was tentatively discarded because low to moderate chitosan concentrations of chitosan produced an increase of the acidification rate of the medium and only high concentrations of the polymer; already producing the cell disruption resulted in a decrease of the acidification rate of the medium. The first possibility could be tested by analyzing the effects of the polymer on the uptake of ^86^Rb^+^. If chitosan produces the reversible displacement of the monovalent cation from the surface, its effect should be reflected in an increase of the Km for its transport, but no change in its *V*_max⁡_ Results of the experiments showed that low amounts of chitosan (20 *μ*g·mL^−1^/12.5 mg of cells) produced a clear inhibition of Rb^+^ uptake (Table 1). Variability of data for *V*_max⁡_ was large, not so for Km, which was found to increase by the addition of this concentration of chitosan without any significant change of  *V*_max⁡_. As expected, this polymer concentration produced an inhibition close to the competitive type, that is, an increase of apparent Km and no change of  *V*_max⁡_.

**Figure 5:**
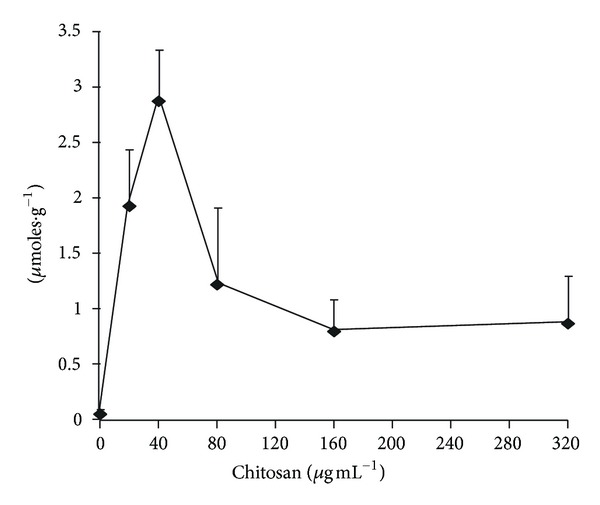
Effects of chitosan on ^45^Ca^2+^ uptake by *Candida albicans* strain ATCC 10231. Starved cells (12.5 mg, wet weight) were added to 10 mM MES-TEA, pH 6.0, and 20 mM glucose and concentrations of chitosan as indicated, in 0.5 mL final volume, in a water bath at 30°C. After 2 min, 50 *μ*M ^45^CaCl_2_ was added, and after two more minutes of incubation, aliquots of 0.1 mL were filtered through a 0.25 m nitrocellulose filter and washed twice with 0.5 mL of 10 mM CaCl_2_. The filters were then dried and placed in vials with scintillation liquid and counted in a Beckman scintillation counter.

In the study of amiodarone, another agent that also produces K^+^ efflux from yeast cells [19–21], it was found that parallel to the increased plasma membrane potential produced by the drug, a several fold increase of  ^45^Ca^2+^ uptake takes place. In a similar experiment with *C. albicans*, we found that, as expected from the hyperpolarization of the cells, low concentrations of chitosan produced a remarkable increase of the uptake of the divalent cation; however, higher concentrations, presumably by producing the disruption of the plasma membrane resulted in a large decrease of the uptake (Figure 5). However, the decrease of the divalent cation uptake did not reach zero.

### 3.6. Effects of Chitosan on Cell Viability and Growth

**Figure 6:**
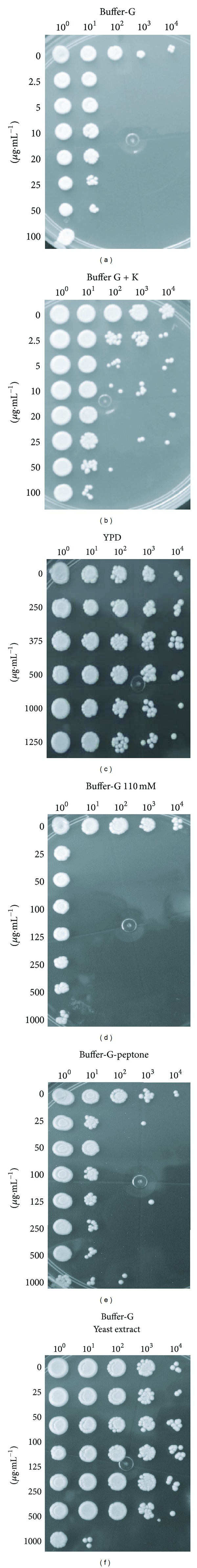
Viability of *C. albicans*, strain ATCC 10231 after incubation with variable concentrations of chitosan under different conditions. Nonstarved cells (2.5 mg) and successive tenfold dilutions in sterile water were incubated in 96-well plastic plates as described under experimental procedures for 10 min at 30°C in 10 mM MES-TEA buffer, pH 6.0 plus (a) 20 mM glucose; (b) 20 mM glucose and 10 mM KCl; (c) YPD medium only; (d) 110 mM glucose; (e) 20 mM glucose and 2% bactopeptone; (f) 20 mM glucose and 1% yeast extract, and each one with variable amounts of chitosan in *μ*g·mL^−1^ as indicated, in a final volume of 0.2 mL. Then, they were spotted using a solid pin multiplate replicator in solid YPD medium. Images were captured after 48 h of growth.

The experiments to analyze the effects of chitosan on cell viability showed that spotting dilutions (with a solid pin multiplate replicator) of the cell suspension after incubation with very small amounts of chitosan in simple buffer (between 2.5 and 5.0 *μ*g·mL^−1^ added to the incubation mixture) caused a clear decrease of growth (Figure 6(a)). However, the effect clearly depended on the amount of cells. At the higher cell concentration, the cells grew at all concentrations of chitosan, up to 100 *μ*g·mL^−1^. This was also observed at the first tenfold dilution of the cells. However, at the highest cell dilutions, growth was inhibited even after incubation with 2.5 *μ*g·mL^−1^. Experiments with other polycationic molecules [3] have demonstrated that their effects can be inhibited by KCl; in the presence of 10 mM KCl in the incubation medium, a very small protection was seen (Figure 6(b)). However, in those cells incubated in YPD, chitosan showed a very small effect; some growth was observed even at the highest dilution of the cells (10^4^), 1.25 mg·mL^−1^ of chitosan (Figure 6(c)). To elucidate which component of the YPD medium conferred “protection” against chitosan, the cells were also incubated in MES TEA 10 mM, pH 6.0 with 110 mM glucose, or with 2% bactopeptone and 20 mM glucose, or with 1% yeast extract and 20 mM glucose. Increasing the glucose concentration to 110 mM (the concentration in YPD) did not result in any significant reversal of the chitosan effect, but on the contrary, growth inhibition was more evident (Figure 6(d)). Bactopeptone produced some reversal of the chitosan effect (Figure 6(e)). The incubation in the presence of yeast extract resulted in almost the same growth as with YPD (Figure 6(f)).

**Figure 7:**
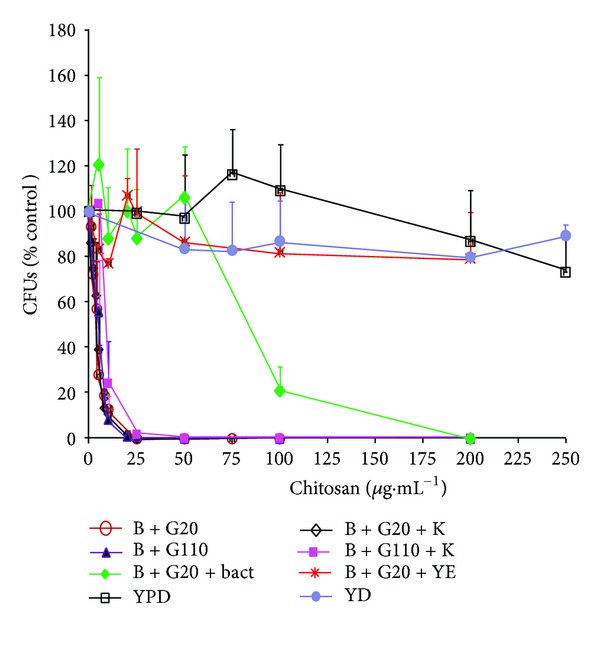
Colony forming units of *C. albicans* strain ATCC 10231 after incubation with variable concentrations of chitosan under different conditions. Nonstarved cells (10 mg wet weight) were incubated for 10 min at 30°C in YPD, or 10 mM MES-TEA buffer, pH 6.0 plus the indicated additions in a final volume of 1 mL with the indicated chitosan amounts. Then, 5 *μ*L of a 10,000-fold dilution was spread in plates of solid YPD medium, and colonies were counted after 48 h. Tracings of incubations as indicated in the chart with buffer (B) plus: ○: 20 mM glucose (G 20); *◊*: 20 mM glucose and 10 mM KCl (K); ▲: 110 mM glucose (G110); ■: 110 mM glucose and 10 mM KCl; *◆*: 20 mM glucose and 2% bactopeptone (Bact); ×: 20 mM glucose and 1% yeast extract (YE); and □: YPD; ●: YD.

The results of the CFU experiments are shown in Figure 7: (a) when the cells were incubated with chitosan in buffer; low concentrations already decreased the number of colonies; (b) in the presence of 10 mM KCl, only a tendency was observed, which was insignificant because of the large standard deviations, in the sense that for a similar decrease of the number of colonies, slightly higher concentrations of chitosan were required; (c) when the cells were incubated in buffer with glucose and yeast extract or with YPD, they resisted much higher concentrations of the polymer; (d) with bactopeptone, the cells resisted up to 50 *μ*g·mL^−1^; that is, 10-fold higher concentrations of chitosan than those effective only with buffer.

**Figure 8:**
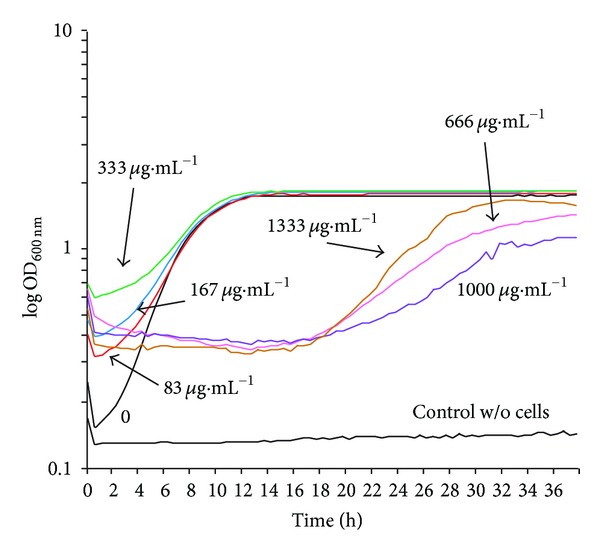
Growth curves of *C. albicans* strain ATCC 10231 with different concentrations of chitosan. The cells (0.03 final optical density units at 600 nm) were placed into 300 *μ*L of YPD in the presence of indicated chitosan concentrations and growth was followed for 40 h.

Growth curves in YPD (Figure 8) showed that chitosan only inhibited growth starting at 666 *μ*g·mL^−1^ or more. Besides, even at higher concentrations, after several hours, growth inhibition recovered.

### 3.7. Chitinase Assay

**Figure 9:**
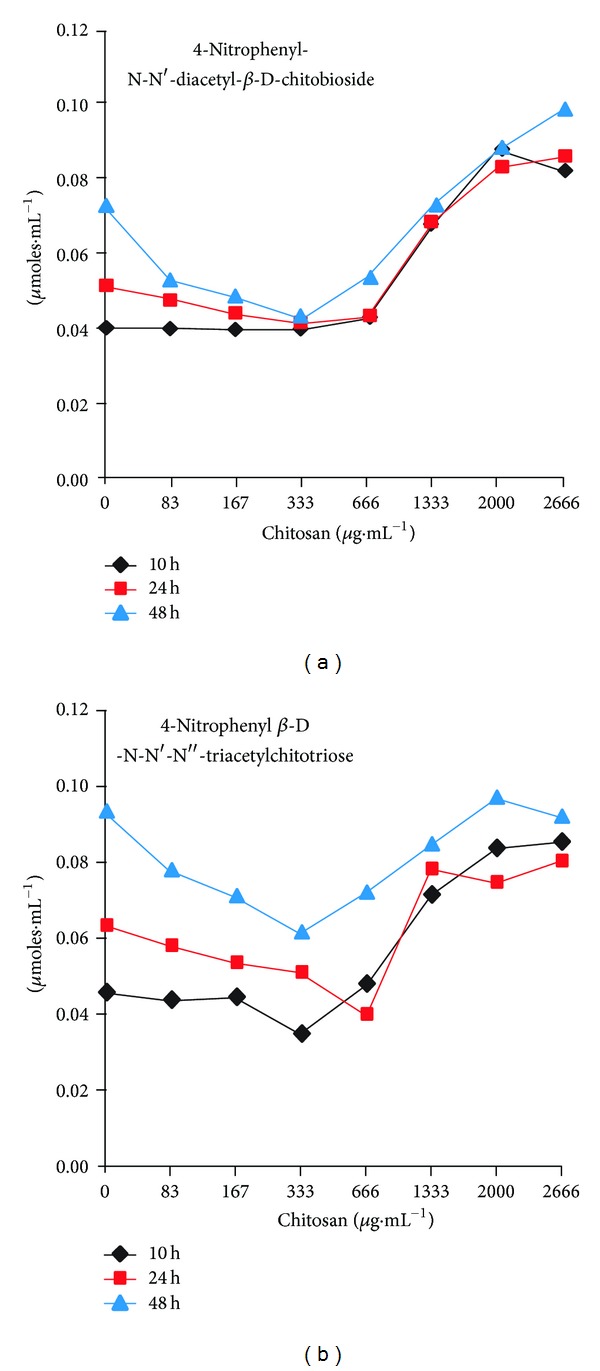
Chitinase activity after long incubations in YPD. Chitinase was measured with 4-nitrophenyl-N-N′-diacetyl-*β*-D-chitobioside and 4-nitrophenyl *β*-D-N-N′-N′′-triacetylchitotriose as substrates. Samples were taken from experiments similar to those for the growth curves with different concentration of chitosan, at three different times of growth, as indicated. Activity was measured in aliquots of the supernatants as described under experimental procedures.

Growth curves in YPD showed that after 16 h, at high concentrations of chitosan, the cells started to grow; so, we considered that the cells might be degrading chitosan and decided to measure the activity of chitinase in the supernatants of experiments performed as for the growth curves at different times. Chitinases show activity on chitosan [22] and most of its activity produced in yeast cultures (90%) is secreted into the growth medium when cells are grown in a nutrient-rich medium, YPD [23]. With the two exochitinase substrates of the assay kit, we observed first an increase of the activity with time in the absence of chitosan; however, another important increase of the activity at higher chitosan concentrations was observed (Figure 9). Results with the exochitinase substrates presented provide information about exosplitting activity on the polymer chains, as it happens on higher analogs of chitin [24, 25]. Even though chitosan is deacetylated, chitinases can degrade it [22], and chitosan degradation by chitinases could explain why cells resumed growth after a long period of time at high concentrations.

### 3.8. Effects on Oxygen Consumption and Fermentation

**Figure 10:**
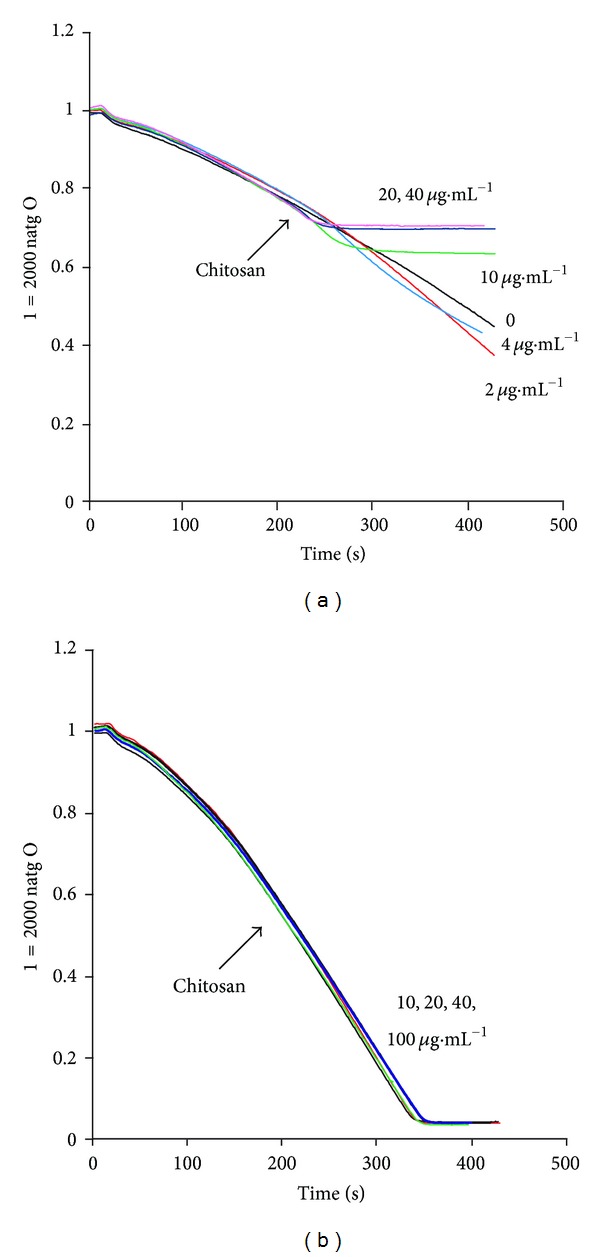
Effects of chitosan on *C. albicans* ATCC 10231 respiration under different conditions. Cells (25 mg, wet weight) were added to 10 mM MES-TEA buffer, pH 6.0, and 20 mM glucose in a final volume of 5.0 mL. Chitosan (*μ*g) was added at the indicated times and concentrations (a). In (b), a similar experiment was performed in the presence of 20 mM potassium phosphate, 1.4 mM NAD^+^, 1.8 mM ATP, and 5 mM MgCl_2_.

Since the K^+^efflux produced by chitosan appears to deplete the cells of K^+^ and other small molecules, it was decided to find out what effect this might have on the main metabolic pathways of the cells, that is, respiration and fermentation. It was found that very low concentrations of chitosan (2 *μ*g·mL^−1^) stimulated respiration, but already 10 *μ*g·mL^−1^ completely inhibited it (Figure 10(a)). It must be noted that in these experiments, in order to be able to follow the oxygen consumption rate, a lower concentration of cells than that in other experiments had to be used (5 mg·mL^−1^).

Considering that inhibition of respiration might be due to the efflux of small molecules (Figure 10(a)); in another experiment, the effect of the polymer at different concentrations was tested but adding also to the incubation medium a mixture of NAD^+^, potassium phosphate, ATP, and MgCl_2_, attempting to compensate for their efflux. It was found that in the presence of these metabolites, respiration was preserved (Figure 10(b)). Moreover, its sensitiveness to CCCP was recovered, indicating the integrity of the mitochondrial function (not shown). The addition of phosphate-TEA alone (from 5 to 50 mM) did not prevent the inhibition of respiratory activity produced by chitosan (not shown).

**Figure 11:**
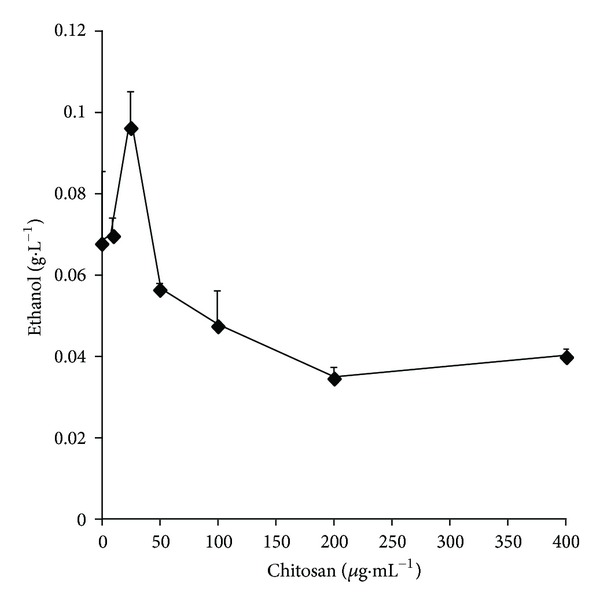
Effects of variable concentrations of chitosan on fermentation of the strain *C. albicans* ATCC 10231. The incubation medium was 10 mM MES-TEA, pH 6.0, and 20 mM glucose in a final volume of 1.0 mL to which 12.5 mg of fasted cells (wet weight) was added. After incubation at 30°C in a water bath for 10 min, the cells were cooled in ice water and centrifuged. Ethanol was measured in aliquots of the supernatants as described under experimental procedures.

Since this species has a significant fermentation capacity [15]; experiments were performed incubating the cells with variable concentrations of chitosan. As Figure 11 shows, chitosan at low concentrations (10 to 25 *μ*g·mL^−1^) stimulated fermentation and inhibited it at higher ones (50 *μ*g·mL^−1^).

### 3.9. Chitosan Binding

**Figure 12:**
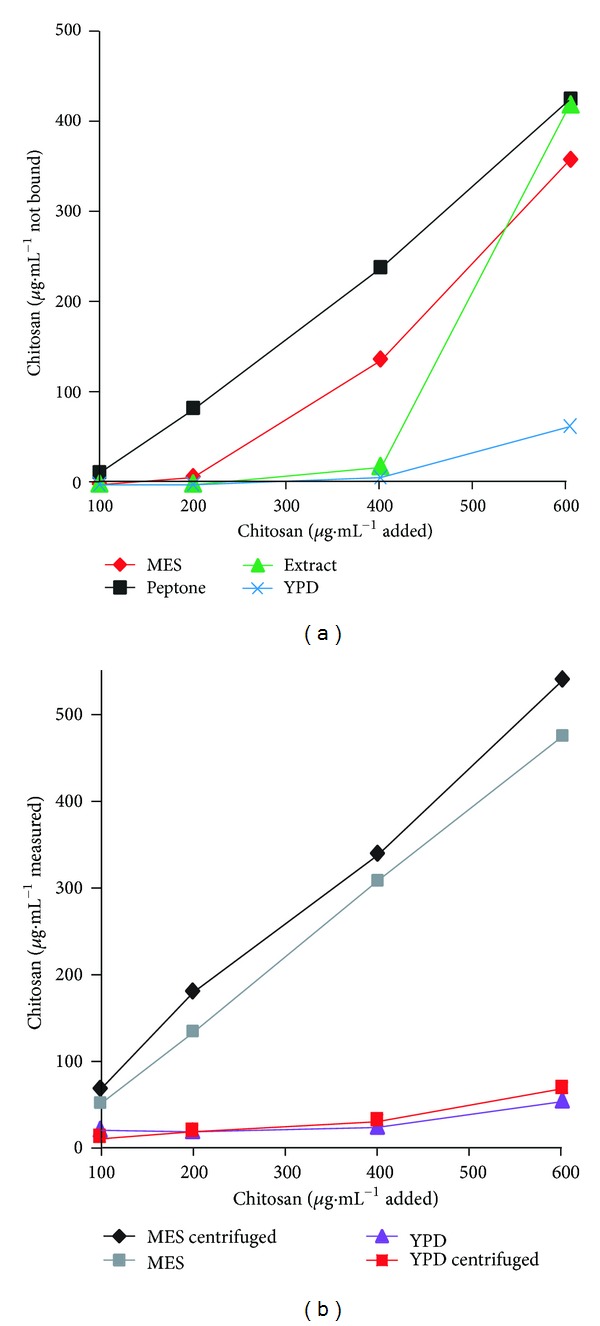
Chitosan binding to *C. albicans* ATCC 10231 at different concentration ratios of the polymer. Cells (12.5 mg·mL^−1^) were incubated in 10 mM MES-TEA buffer, pH 6.0, and 20 mM glucose; or 20 mM glucose and 2% bactopeptone; or 20 mM glucose and 1% yeast extract or YPD medium only; each one with variable amounts of chitosan in *μ*g·mL^−1^ as indicated, in a final volume of  1 mL for 10 min at 30°C. Then, they were centrifuged (1625 g) and the remaining chitosan in the supernatant was measured by means of its fluorescence change in bicine-TEA 10 mM pH 8 with 10 *μ*M calcofluor at 350–440 nm (a). Another experiment was performed without cells, with or without centrifugation (b).

The finding that the effects of similar amounts of chitosan were larger as the amount of cells was decreased, suggested its strong binding to the cells. The experiments performed to measure this parameter in fact demonstrated that this was the case; free chitosan was found only when the cell/polymer ratio was large. Having found that chitosan effects on growth and viability were smaller in YPD, we expected that binding in this incubation medium would decrease; what we found was that the free polycation in the medium did not increase but decreased. (Figure 12(a)). So, we did the same experiment but without cells, finding that chitosan precipitates with YPD, and centrifuging it or not, the fluorescence produced with calcofluor is quenched, indicating an interaction of chitosan with the YPD components (Figure 12(b)). 

## 4. Discussion

About the possible use of chitosan as an antimycotic, Rabea et al. [2] stressed the following: “Chitosan is inexpensive and nontoxic and possesses reactive amino groups. It has been shown to be useful in many different areas as an antimicrobial compound in agriculture, as a potential elicitor of plant defense responses, as a flocculating agent in wastewater treatment, as an additive in the food industry, as a hydrating agent in cosmetics, and more recently as a pharmaceutical agent in biomedicine.” However, these authors also stress the following: “… it is a current matter of discussion as to whether these biopolymers may have the potential to influence physiological functions or metabolism in the microorganisms. Therefore, a significant increase in the number of scientific studies to obtain evidence to support this can be expected.”

It is well known that chitosan may be an effective fungicidal, but it has been found that rather high concentrations are required to inhibit yeast growth [2, 6–8, 12, 13, 26–28]. However, the experiments reported here show a series of interesting facts regarding its effects at low to intermediate concentrations, even though under those conditions and even after incubation in a simple buffered medium, the polycation did not affect cell viability. Besides, we found that the effects depend on the incubation medium because of a series of factors.

When the cells were incubated in simple buffer, under conditions not very different to those employed by Courchesne [19] and by Maresova et al. [20], these authors proposed, analyzing the effects of amiodarone, that the increase of the internal Ca^2+^ concentration may kill the cells by an apoptosis-like mechanism. Working with this same compound, we proposed a mechanism [21] that may also be valid for chitosan as follows.

1. The strong binding of chitosan, a polycationic molecule, decreases the negative surface charge of the cells (to more positive values), as shown by the decreased mobility of the cells under an electric field.
2. This change decreases the actual concentration of cations at the surface, mainly K^+^, altering the steady state balance between its internal/external concentration and the plasma membrane electric potential difference, resulting in its efflux. In fact, it is known that the surface charge of the cells clearly can influence the kinetic properties of monovalent cation uptake [29]. The experiments performed with ^86^Rb^+^ showing an increased Km without a change of  *V*_max⁡_ indicate that in fact, and possibly due to the decreased effective concentration of the cation at the cell surface, higher concentrations were required to achieve half the maximal velocity of the uptake.
3. This efflux then gives rise to a hyperpolarization of the plasma membrane. This could be demonstrated by the estimation of electric plasma membrane potential difference with DiSC_3_(3). We use the term efflux (carrier mediated) and not leakage resulting from the permeabilization of the membrane, because although both mechanisms would increase K^+^ exit and decrease the uptake of Rb^+^, leakage hardly would allow the cells to reach a similar *V*_max⁡_ value in the presence of chitosan. It is also difficult to imagine an increase of the electric plasma membrane difference resulting from the cation leakage through a damaged membrane. The possible efflux pathways may be the same K^+^ transporter, or the equivalent to Tok1p which has been at least identified in *Candida glabrata* [30].
4. This hyperpolarization of the plasma membrane results in a remarkable increase of Ca^2+^ uptake, as well as the efflux of negatively charged molecules, such as phosphate, nucleotides, and substrates of enzyme reactions.

It is quite possible that the apoptosis-like mechanism proposed by Courchesne [19], Maresova et al. [20], and Muend and Rao [31] is part of the fungicidal mechanism of chitosan, but also that the efflux of nucleotides and substrates of the metabolic pathways results in the inhibition of the main metabolic pathways, respiration, and fermentation depriving the cells of their main energy sources.

The large increase of Ca^2+^ uptake observed at low concentrations of chitosan, which appears to be due to the membrane hyperpolarization, is most interesting and would deserve further investigation regarding this remarkable effect of the hyperpolarization of the plasma membrane on the uptake of the cation. This is different from previous work on the effects of amiodarone on *S. cerevisiae*. This drug, which also induces the efflux of K^+^ and an increase of the plasma membrane electrical potential difference, without apparently disrupting the plasma membrane, produces a remarkable increase of  ^45^Ca^2+^ uptake that does not decrease at the highest concentrations used [21]. Besides, both with amiodarone and moderate chitosan concentrations, it is possible that the Ca^2+^ taken up is stored in the vacuole or other intracellular compartment. Even though the high chitosan concentrations appeared to permeabilize the plasma membrane, there was always a significant amount of the divalent cation remaining in the cells, probably within the vacuole.

Although this basic proposal appears to be valid for the mechanism of action of chitosan as an antifungal, there are other factors involved, on which the effects depend. It is clear that the effects of chitosan vary with the concentration or amount of cells used in each experiment. One illustrative experiment in this respect was that in which K^+^ efflux was measured using different cell concentrations (Figure 1(c)). It was evident that the effect was proportionally higher at the low cell concentration, which, on the other hand, can be explained because of the strong binding of the polymer to the cells.

The experiments also showed that the effects of chitosan also depend on the medium used; it was clear that the effects on viability were lowest in YPD. Dissecting the YPD components, the glucose inhibition was already observed by Granot et al. [32]; glucose in the absence of other necessary nutrients is accompanied by rapid production of ROS, which does not occur in the complete medium (YPD). Hoeberichts et al. [33] observed that glucose-induced cell death correlates with K^+^ and nucleotides effluxes and can be explained if H_2_O_2_ activates plasma membrane proteins mediating the efflux of these small molecules. K^+^ efflux is concomitant with the nucleotide release from the cells. However, what has been reported requires longer incubation times than those employed by us.

With the other components of YPD medium, it was found that yeast extract was also responsible for a higher viability of the cells, most probably because of its salt content (calcium, phosphate, chloride, potassium, sodium, and others). As a simple approach to this, the effects of 10 mM KCl were tested, with a low protection against the damage produced by chitosan, which was observed when spotting different cell dilutions on solid plates, although only as a tendency in the CFU experiments, due to the large standard deviation values. This effect was small, compared to that reported by Yphantis et al. [3] on the efflux of nucleotides under the action of other polycations, most probably because the binding of chitosan to the cells is much stronger than that of the polymers used by those authors. Also anions may complex chitosan, as shown by the decrease of free chitosan in the presence of YPD and in agreement with Young et al. [11]. These facts may be the reason why growth inhibition, as observed in the three methods to analyze viability and growth was greatly diminished when incubation was performed in YPD or yeast extract, or even bactopeptone. This is important in relation with the possible use of chitosan as an antifungal, since its effect may be influenced mainly by the salt (anions and cations) concentration of the surrounding medium; in fact, in most reported antimicrobial effects, the effective concentrations are around or higher than 1 mg·mL^−1^ [2].

Other effects of chitosan have been described, among them the inhibition of the H^+^-ATPase activity; found and studied in *C. albicans* by Gupta et al. [34]. In the plant *Mimosa pudica,* the proposed primary site of action of chitosan is the H^+^-ATPase, that is, an inhibition of proton pumping [27]. It has also been found that chitosan inhibits the H^+^-ATPase of *Rhizopus stolonifer*, but this occurs at very high concentrations of the polycation [35]. However, in the present work, proton pumping measured as the capacity of the cells to acidify the medium simultaneously to K^+^ efflux to the incubation medium, at low chitosan concentrations was found increased. This may be due to the increased fermentation rate also observed at low chitosan concentrations, resulting in an increased production of CO_2_, which hydrates to H_2_CO_3_, increasing the acidification of the medium. 

### 4.1. Effects at Higher Concentrations

At higher concentrations, independently of the medium used, the effects of the polycation appear to be much more profound but in summary resulting in the disruption of the cell membrane with: (a) the subsequent efflux of all small molecules capable of traversing the cell wall, such as those studied herein, phosphate, nucleotides, and, of course, K^+^; (b) the loss of metabolic capabilities, as shown by the effects on respiration and fermentation, also due to the loss of at least phosphate and nucleotides; (c) the efflux or inability to take up Ca^2+^ because of the cell membrane damage, and (d) the inhibition of growth. 

It is interesting in this respect that, even when the cell membrane is damaged, proteins are not able to cross the cell wall, as seen in the experiments with glucose-6-phosphate dehydrogenase and as reported before with amiodarone in *S. cerevisiae* [21]. Also the experiments in which respiration was restored by the addition of phosphate, ATP, and NAD^+^ indicate that even if the cell membrane is damaged, the internal structures (vacuole, mitochondria, and other vesicles) are preserved, and proteins remain inside the cell. This fact may provide another way to permeabilize the cells and to study their metabolism *in situ* without preparing protoplasts, as required by other methods [5, 36]. Measurement of glucose-6-phosphate dehydrogenase and respiration are examples of the study of an enzyme or an organelle *in situ*, by adding the necessary cofactors. We also found that in the presence of an isosmotic sorbitol concentration, an important part of the phosphates remained in the cells, probably compartmentalized in the vacuole. Results of these experiments open the possibility to study chitosan as a suitable agent to study compartmentalization of solutes within the cell, similarly to diethylaminoethyl-dextran [5]. 

We also found that after 16 h in the presence of high chitosan concentrations, when the cells recover and start growing, the activity of chitinases was increased. It is interesting that even in the absence of chitosan at short times, both enzyme activities increased with time and were decreased with chitosan. However, at longer times, they showed a larger increase with the increasing chitosan concentrations. Yeasts and yeast-like fungi have several chitinases, *S. cerevisiae* has two, and *C. albicans* has four [37]. This is an additional fact to be considered regarding what appears to be a recovery or resistance mechanism of the cells to already high concentrations of the polymer, even when damage produced appears to be quite profound.

Fermentation and respiration were stimulated by low chitosan concentrations, probably because of the energetic demand to contend with what appears to be a moderate insult to the cells, in agreement with Palma-Guerrero et al. [38]. Then, at high chitosan concentrations, due to the permeabilization of the membrane, fermentation is inhibited because of the loss of glycolytic intermediaries and cofactors.

Our experiments revealed another interesting fact: the fungicidal capacity of chitosan required much higher concentrations when the incubation medium was YPD. We so believed that YPD, mainly because of the salt content of the yeast extract should block the binding of chitosan to the cells. The experiments to measure this parameter showed that the binding of chitosan was similar when the cells were incubated with the polymer in simple buffer or in YPD, indicating that the decreased effect of chitosan in YPD or yeast extract was not due to a decreased binding to the cells as a whole. However, the experiments in which chitosan was incubated with YPD without cells show that it flocculates and precipitates the polymer, avoiding or at least decreasing its binding to the cells. At much longer incubation times, as those used in the growth curves with chitosan concentrations that do not kill the cells, chitinase activity may also decrease its concentration.

**Figure 13:**
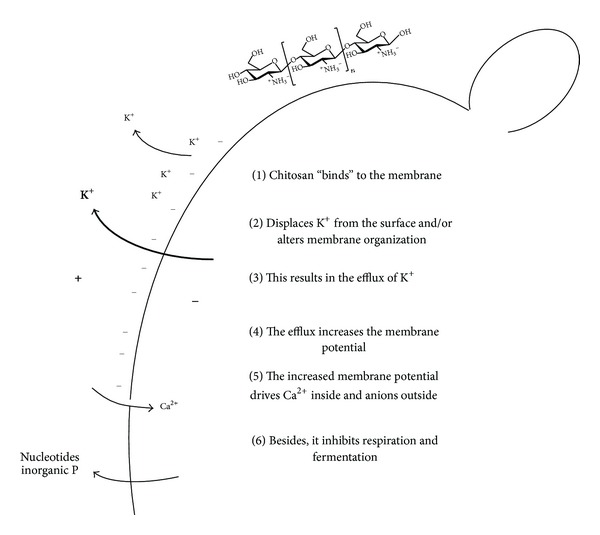
Postulated action mechanism of low to medium concentrations of chitosan. (1) Chitosan, because of its positively charged amino groups binds to the surface of the cells; (2) this produces the displacement of the K^+^ attached to the surface, disrupting the equilibrium between the electric plasma membrane difference and the relative internal/external concentrations of the cation; (3) this loss of equilibrium results in the efflux of K^+^; (4) the efflux of positively charged potassium ions results in an hyperpolarization of the plasma membrane; (5) the hyperpolarization of the plasma membrane greatly increases the uptake of Ca^2+^ and the efflux of negatively charged small molecules, and (6) metabolic functions as fermentation and respiration are also affected.

We expect that our present results are a significant contribution to the knowledge about the effects of low chitosan concentrations, summarized in Figure 13, which are consistent with our former proposal for the effects of amiodarone.

### 4.2. Other Strains or Species

Finally, although the experiments presented were performed with *C. albicans* strain ATCC 10231, most experiments were repeated with similar results with the strain ATCC 90028 and with *C. dubliniensis* strain CD 36 (not shown), indicating first that the effects are not particular to one strain but general to other *C. albicans* strains or even *Candida* species, *S. cerevisiae*, and probably many other yeast cells or even fungi. With all these strains or species, we found the efflux of K^+^, the inhibition of respiration and fermentation at moderate to high concentrations, the plasma membrane hyperpolarization, the remarkable increase of Ca^2+^ transport at low concentrations and its decrease at higher concentrations, and the requirement of rather high concentrations of chitosan to inhibit growth in YPD medium. In fact, there are many reports showing the antifungal capacity of this polymer against many other fungi and bacterial species [2, 6–8, 12, 13, 26–28].

## 5. Conclusion

Even though chitosan has been considered essentially as a permeabilizing agent for bacteria, fungi, and plant cells, our results show a series of alterations of ion homeostasis and metabolism which occur at concentrations lower than those required to kill the cells.

At high concentrations, the main effect is permeabilization, so chitosan may be suitable to study compartmentalization of solutes, enzymes, and metabolic functions *in situ*.

Because of all factors affecting its fungicidal effects, regarding its use as an antifungal, we would recommend its use at concentrations higher than 1.0 mg·mL^−1^. This may assure a fungicidal and not only a fungistatic action.

## Figures and Tables

**Table 1:** Kinetic constants of ^86^Rb^+^ uptake; effect of chitosan on the strain *C. albicans* ATCC 10231. Uptake of ^86^Rb^+^ was measured incubating 12.5 mg of starved cells in 10 mM MES-TEA, pH 6.0, and 20 mM glucose, 0.5 mL final volume, in the absence or presence of chitosan, 20 *μ*g·mL^−1^ plus the indicated amounts of chitosan in a water bath at 30°C. After 2 min, 0.1 to 0.6 mM of ^86^RbCl was added. After two more minutes, aliquots of 0.1 mL were taken which were filtered through a 0.25 *μ*m nitrocellulose filter and washed twice with 0.5 mL of 10 mM CaCl_2_. The filters were then dried and placed in vials with scintillation liquid and counted in a Beckman scintillation counter (*n* = 7).

	Km, mM	*V*_max⁡_, *μ*Eq·g^−1^ in 2 min
Control	0.41 ± 0.08	8.7 ± 1.24
Chitosan	2.30 ± 0.05	8.9 ± 3.8

## Abbreviations

CCCP: Carbonyl cyanide 3-chlorophenylhydrazone
CFU: Colony forming units
DiSC_3_(3): 3,3′-Dipropylthiacarbocyanine or cyanine
MES-TEA: 2-(N-Morpholino) ethanesulfonic acid, adjusted to pH 6.0 with triethanolamine
TEA: Triethanolamine.
